# Effects of Ovariectomy and Low-Calcium Diet on Six Different Sites of the Rat Skeleton

**DOI:** 10.3390/biomimetics10070474

**Published:** 2025-07-18

**Authors:** Xanthippi Dereka, Rodopi Emfietzoglou, Pavlos Lelovas

**Affiliations:** 1 Department of Periodontology, School of Dentistry, National and Kapodistrian University of Athens, 2 Thivon Str, 115 27 Athens, Greece; remfiet@dent.uoa.gr; 2 Laboratory of Experimental Surgery and Surgical Research “N.S. Christeas”, School of Medicine, National and Kapodistrian University of Athens, 115 27 Athens, Greece; paulveterin@yahoo.com

**Keywords:** ovariectomy, low-calcium diet, rat skeleton, micro-architecture, biomimetics

## Abstract

The aim of this study was to evaluate structural and micro-architectural changes in the mandible, parietal bone, femur, and tibia in OVX rats at different time periods after ovariectomy. Forty-two 11-month-old female Wistar rats were used. Six rats without surgery were euthanized to serve as a baseline. Eighteen rats were ovariectomized and fed with a calcium-deficient diet, and eighteen animals were used as controls (Ctrls) and fed with a standard diet. Six OVX rats and six Ctrls were euthanized at 3, 6, and 9 months. Qualitative histology and dual-energy X-ray absorptiometry (DXA) were performed. Histological evaluation of bones harvested from the OVX groups revealed trabecular bone reduction, while no significant differences in the cortical bone of OVX and Ctrls were observed. DXA measurements of (1) femoral diaphysis showed a significant decrease in the OVX group compared to the Ctrl groups at 3 (*p* = 0.041), 6 (*p* < 0.001), and 9 months (*p* < 0.001); (2) the proximal tibia showed a significant decrease in the OVX group compared to the Ctrl groups (*p* < 0.001); (3) parietal bone showed a significant difference between OVX and Ctrls at 6 months (*p* = 0.012); and (4) the mandible showed no significant differences between the OVX and Ctrl groups. OVX aged rats might present reductions in the density of the femoral diaphysis, proximal tibia, parietal bone, and mandible at different time points. These findings contribute to the field of biomimetics by providing more details for the understanding of age- and hormone-related bone changes in the osteoporotic-like rat model. Such data are critical for the development of biomimetic materials and structures that attempt to simulate natural bone adaptation and deterioration, especially in the context of postmenopausal or osteoporotic conditions.

## 1. Introduction

Osteoporosis is one of the most common skeletal disorders, mainly affecting postmenopausal women [1,2]. Being associated with loss of bone mass and subsequent changes in bone micro-architecture, the disease increases patients’ susceptibility to fractures [3]. Osteoporosis may manifest in craniofacial structures, causing reductions in the area of cancellous bone and in the number and thickness of trabecular plates [4,5,6]. Some studies have suggested that osteoporotic patients present increased alveolar ridge resorption [7,8], while other studies failed to verify this finding [9,10].

In today’s aging societies, the number of patients with osteoporosis requiring oral rehabilitation with dental implant-supported prostheses or bone regeneration techniques for the treatment of bone defects is increasing. Special consideration is required when performing such treatments, because the disease or its therapy may affect wound healing, bone regeneration, and implant-associated outcomes [11,12].

The successful rehabilitation of osteoporotic patients with dental implants—and, if necessary, bone regeneration procedures—requires profound comprehension of the mechanism by which osteoporosis and its treatment affect bone physiology and structure during osseous healing. Research data suggest that although guided bone regeneration may successfully be performed in osteoporotic-like conditions, the formation of less and of poorer-quality new bone should be expected [13,14]. Pre-clinical studies in animal models reproducing the osteoporotic condition may provide valuable insight in the research of bone healing and various therapeutic modalities [15,16]. The increasing interest in biomimetic scaffolds for hard tissue repair underscores the need for well-characterized pre-clinical models that simulate conditions such as postmenopausal osteoporosis and associated bone loss. Moreover, in the field of biomimetics, these experimental osteoporotic models replicating bone remodeling may advance biomimetic approaches to new regenerative materials and therapeutic strategies. The understanding of the pathogenetic mechanism of osteoporosis has helped in the development of drugs for the treatment of the disease, such as strontium ranelate and zoledronic acid. It has been shown that strontium ranelate and zolendronic acid may improve bone formation in critical-size calvarial defects in osteoporotic rats [17,18].

The existing pre-clinical studies differ in terms of study design, methods of osteoporosis induction, and follow-up periods; examine multiple anatomical sites; and, most importantly, use dissimilar animal models of various ages. Taking into consideration the heterogeneity of the available studies, a recent systematic review suggested that osteoporosis may compromise dental implant osseointegration in experimental osteoporotic-like conditions and underlined that the lack of a standardized experimental model constitutes a significant limitation [19].

The design of pre-clinical studies is of principal importance and should aim for the amelioration of research quality and the clinical correlation between animals and humans [20,21]. The ovariectomized (OVX) female rat experimental model has been recommended by the Food and Drug Administration (FDA) because it mimics conditions in postmenopausal osteoporosis and has been widely adopted for bone research [22,23].

Ovariectomy alone may not consistently lead to bone loss in all skeletal sites in rodents, because the resulting changes are site-specific [24]. This limitation can be addressed by combining ovariectomy with a calcium-deficient diet [25,26]. The combination of ovariectomy and a low-calcium diet has been shown to significantly enhance bone loss in ovariectomized rats [27].

A thorough review of the pertinent studies investigating osseous healing during bone regeneration or/and dental implant placement in the OVX rat model raises concerns about the appropriate selection of age, time period after ovariectomy, and sites of the rat skeleton that would present bone changes similar to those of an osteoporotic woman.

The existing studies investigate bone changes in rats who underwent ovariectomy at ages of up to 2 [25,28,29,30,31,32,33], 3 [29,34,35,36,37,38,39,40], 4 [41,42], 6 [24,35,43,44,45,46,47], 11 [35], or even 12 and 14 months [48]. It has been suggested that ovariectomy at the age of 2 months may induce low bone mass, due to inhibited growth, and not actual bone loss, as observed in postmenopausal osteoporosis; meanwhile, non-OVX rats, which serve as control groups, present rapid growth at this age [49]. Meanwhile, Francisco et al. suggested that rats undergoing ovariectomy at the age of 6 months present a true osteoporotic response characterized by decrease in bone mineral and micro-architectural properties and reduced trabecular connectivity [35]. It has been claimed that the cofounding effect of bone growth is minimal in rats undergoing ovariectomy at the age of 6–9 months, which are considered to more closely resemble postmenopausal osteoporosis [50]. Jee and Jao recommended that ovariectomy should be performed at the age of 9 months, because at this age the female rat reaches peak bone mass [51]. Rats undergoing ovariectomy after the age of 9 months present trabecular bone remodeling similar to that of postmenopausal women but, unlike humans, they display a low response of cortical bone in comparison to trabecular bone. Age-related bone loss is observed in rats undergoing ovariectomy after the age of 9 months [52]. Regarding the effects of aging on the mature rat skeleton, Coutel et al. examined 15-month-old rats and suggested that aging does not affect the trabecular bone architecture in the alveolar bone of the mandible or in the tibiae [43]. Various studies have examined bone characteristics at different time periods after OVX, such as 1 [24,29,34,44], 2 [34,46], 3 [21,24,28,48], 3.5 [44], 4 [34,40,47], 5 [38], 6 [24,32], 9 [24], or even 12 months [45]. Various studies have examined the effects of ovariectomy on the tibia and femur, while limited publications have investigated the effects of OVX on the mandible and parietal bone, which are commonly used in the research of various biomaterials and osseous healing [14,18,24,53,54,55,56].

The hypothesis of this study was that OVX rats would present different degrees of changes in the structure and architecture of the alveolar bone in the mandible, parietal bone, femur, and tibia, and that these changes would progress with age. The comparison of these changes, evaluated by histological observation and dual-energy X-ray absorptiometry (DXA), will provide information (a) for the understanding of age- and hormone-related bone changes in this experimental rat model, and (b) on the most appropriate site and age of rats for the study of bone regenerative techniques and dental implant placement. Furthermore, the findings might contribute to the field of biomimetics, since such data are essential for the development of biomimetic materials and structures that will more closely resemble human bone adaptation and degeneration, especially in the context of postmenopausal or osteoporotic conditions.

Thus, the aim of this study was to evaluate the structural and micro-architectural changes in the mandible, parietal bone, femur, and tibia in OVX rats at different time periods after ovariectomy.

## 2. Materials and Methods

### 2.1. Experimental Animal Model and Induction of Osteoporosis-like Condition

The protocol was prepared before the study to determine the parts of the rat skeleton that would be investigated and the time points that would be studied.

The present study was evaluated by the research establishment’s Protocol Evaluation Committee and was approved by the General Directorate of Veterinary Services (permit no. 588) according to Greek legislation (Presidential Decree 56/2013, in compliance with the Directive 2010/63/EU). The ARRIVE guidelines for reporting animal research were followed. This study also followed the 3Rs principle, as well as national guidelines for the proper use of animals.

Forty-two 11-month-old female Wistar rats weighing between 203.6 and 315.6 g at the beginning of the study were used. The animals were allowed to acclimatize for two weeks, they were kept at constant room temperature (22 °C) with a 12 h day/night cycle and ad libitum access to drinking water, and they were fed according to the diet of the group to which they were assigned. The animals were subsequently randomly assigned to the groups (each animal received a random number from 1 to 42 and, through random number generator software, the animals were allocated into groups) (week 0). The technician who allocated the cages into ranks was blinded for the groups. The only person who was aware of the groups of the animals was the principal investigator, while the researchers performing the DXA measurements and the statistical analysis were blinded. Six rats without any surgery were euthanized to serve as a baseline (baseline, N = 6). Experimental osteoporosis was induced in eighteen randomly selected rats by bilateral ovariectomy and a calcium-deficient diet containing 0.1% calcium and 0.77% phosphorus (OVX, N = 18). The remaining eighteen animals were used as healthy controls and fed with a standard diet containing 1.1% *w*/*w* calcium (Ctrl, N = 18).

Ovariectomy was performed under anesthesia induced by intramuscular administration of dexmedetomidine (0.025 mg/kg) and ketamine (50 mg/kg). The animals were placed in a dorsally recumbent position, and the fur on their abdomens was clipped. The exposed skin was disinfected. Antibiotics and analgesia (enrofloxacin (5 mg/kg) and carprofen (4 mg/kg), respectively) were administered subcutaneously. Entrance to the peritoneal cavity was achieved through a midline ventral incision at the linea alba, at a length approximately one-third of the distance between the xiphoid and prepubic processes. The vessels of the ovarian plexus and the ovarian ligament were ligated, and the ovaries were excised. The remaining part of the uterus was returned to the abdominal cavity. The muscle wall and the skin were closed in layers by single interrupted sutures. After the surgery, anesthesia was reversed for quick recovery with atipamezole, and the animals returned to their home cages.

Six OVX rats and six rats serving as control animals were euthanized at 3, 6, and 9 months of healing (OVX3, OVX6, OVX9, Ctrl3, Crtl6, Ctrl9). The animals were weighed at the beginning of the study, on the day of OVX, and before euthanasia at 3, 6, and 9 months for the OVX and Ctrl groups. Ovariectomy confirmation was performed during the necropsy after euthanasia.

**Figure 1:**
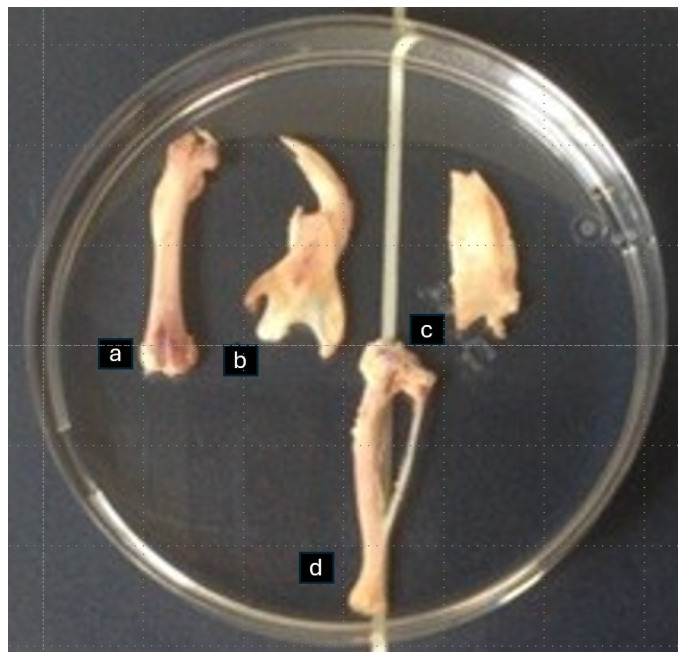
Femur (**a**), half of mandible (**b**), half of parietal bone (**c**), and tibia (**d**) placed in a plastic Petri dish with saline for bone densitometry assessment with DXA.

During the euthanasia, one tibia, one parietal bone, one femur, and one part of the hemisected mandible per animal were harvested. After removal of the soft tissues, the right tibia, right femur, and right half of the mandible and of the parietal bone were placed in a plastic Petri dish with saline for bone densitometry assessment with DXA at the Laboratory for Research of the Musculoskeletal System, School of Medicine, University of Athens, Greece (Figure 1). At the end of this process, the bones were placed in lab vials in a 70% ethanol solution for desalination and histological analysis.

### 2.2. Qualitative Histology Analysis

The tissues were embedded in 5% hydrochloric acid solution for 24 h for decalcification. Then, 5 mm thick tissue samples were obtained from (a) the mandible, in the buccolingual direction, including the area between the roots of the 1st molar and the inferior alveolar duct; (b) the proximal tibia, in longitudinal sections, 1 mm distal to the articular cartilage; (c) the diaphysis of the femur, in longitudinal sections; and (d) the parietal bone, in longitudinal sections. The tissue samples were dehydrated in ethanol and embedded in paraffin. Sections were cut with the microtome set at 5 μm. The specimens were stained with hematoxylin–eosin solution and evaluated with an optical microscope.

### 2.3. Dual-Energy Χ-Ray Absorptiometry (DXA)

**Figure 2:**
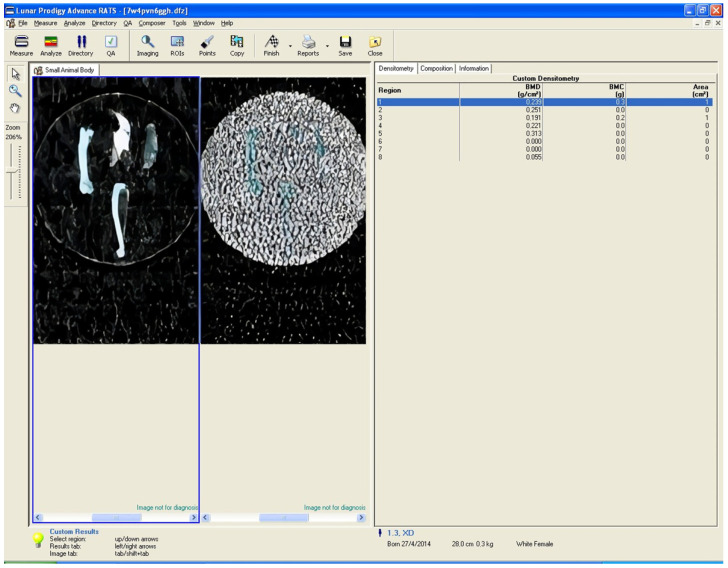
Example of DXA measurement ROI placement for the 6 bone sites (proximal femur, total femur, proximal tibia, total tibia, mandible, and parietal bone).

The DXA method was used to measure bone mineral density (BMD) and bone mass. A GE Lunar Prodigy Densitometer machine equipped with small animal software was used, and specific regions of interest (ROIs) were defined. For the evaluation of the proximal tibia, the ROI was placed in the proximal tibial metaphysis, 3 mm distal to the tibial plateau, while for the assessment of the total tibial BMD, the whole tibia, except for the fibula, was included in the ROI. In the study of the femur, the ROI was placed 5 mm from its distal articular surface, while for the total measurement (total femur) the entire femur was included. For the mandible, the ROI was placed in the middle of the bone, rostral to the roots of the first molar. Regarding the parietal bone, software was used to determine the medial point, and a straight line was drawn (from the rostral to the caudal end of the sample). Its length was calculated, and then a second line, half the length of the first line, was drawn. The ROI was placed in the calculated middle (Figure 2). The in vitro precision (coefficient of variation) of the system was 0.5%. Calibration of the system was performed before each group measurement.

### 2.4. Power Analysis for Sample Size Determination

Power analysis was performed with G Power 3.1 software to estimate the smallest sample size that would yield statistically significant results. For the DXA measurements, the literature was used [57], and the size effect was calculated at d = 2.05. In order to ensure 0.95 power with a significance level of α = 0.05, it was evaluated that the total number of animals needed was 12 in total (6 per group) for each time point. Hence, 36 rats were needed, while 6 additional rats for time 0 served as the baseline group.

### 2.5. Statistical Analysis

Values of continuous variables will be presented using mean values and standard deviations (SDs), with 95% confidence intervals (CIs). Categorical variables will be represented by the frequencies (N) and the corresponding percentages (%). The normality of the data distribution was examined with the Kolmogorov–Smirnov test and a normal probability plot.

A two-way analysis of variance model was used to evaluate the interaction between two variables: intervention (control/OVX) and time (3, 6, 9 months). The comparisons of the absolute values of the variables between the intervention groups (control/OVX) were performed using the independent-samples *t*-test. Welch’s test was used when the variance between the groups was unequal, and the Mann–Whitney test was used if the data were not equally distributed. The longitudinal comparison of variables between the groups was performed using the one-way analysis of variance (ANOVA) model, while the Bonferroni test was used for pairwise comparisons. For data that violated the assumption of homogeneity of variance, the Welch and Games–Howell tests were applied. If the assumption of normal distribution of data was not fulfilled, the non-parametric Kruskal–Wallis and Mann–Whitney tests were performed. Statistical analysis was performed using the SPSS statistical software, version 17.00 (SPSS Inc., Chicago, IL, USA). All of the tests were two-sided. A *p*-value of less than 0.05 was considered to indicate statistical significance, while borderline statistically significant values were described if present (0.05 < *p* < 0.1).

## 3. Results

Two animals (1 Ctrl9, 1 OVX3) died. The subsequent necroscopies revealed no pathological lesion or malignancy upon macroscopic examination. Additionally, no ovarian tissue remnants were detected in the OVX rat, while the uterus was significantly smaller and hypoplastic in comparison to the Ctrl group. The growth of the remaining 40 rats was normal during the study period, and a gradual increase in their weight was recorded. The researcher performing the ovariectomies had great experience in this technique and had never experienced failure; for this reason, there were no humane endpoints.

**Figure 3:**
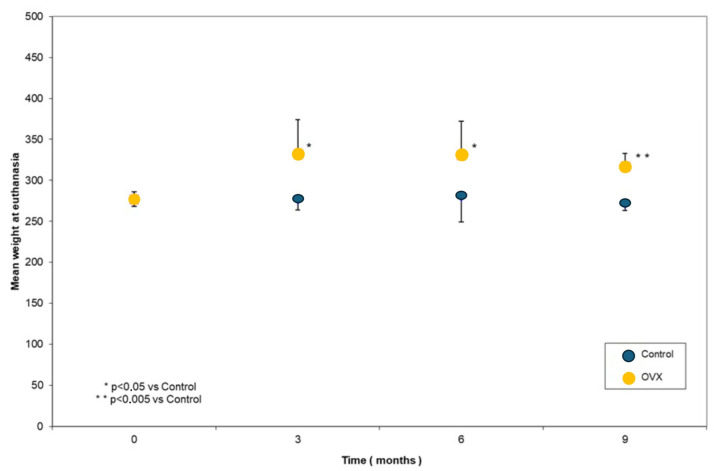
Body weight values at the different time points.

The weight of the OVX animals was significantly increased compared to Ctrls at 3 (*p* = 0.041), 6 (*p* = 0.035), and 9 months (*p* < 0.001). Furthermore, the analysis indicated that the weight of the animals in the OVX3, OVX6, and OVX9 groups was significantly increased in comparison to the baseline group (*p* = 0.001) (Figure 3).

### 3.1. Qualitative Histology Analysis

**Figure 4:**
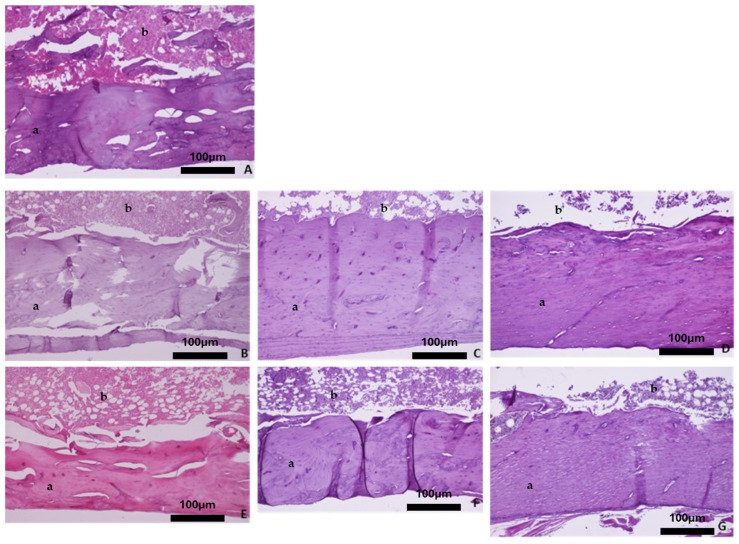
Representative histomicrographs of femurs (hematoxylin–eosin staining; Obj ×10). Trabeculae (a) with part of the bone marrow (b) are depicted. (**A**) Baseline group, (**B**) control group 3 months, (**C**) control group 6 months, (**D**) control group 9 months, (**E**) OVX group 3 months, (**F**) OVX group 6 months, and (**G**) OVX group 9 months.

**Figure 5:**
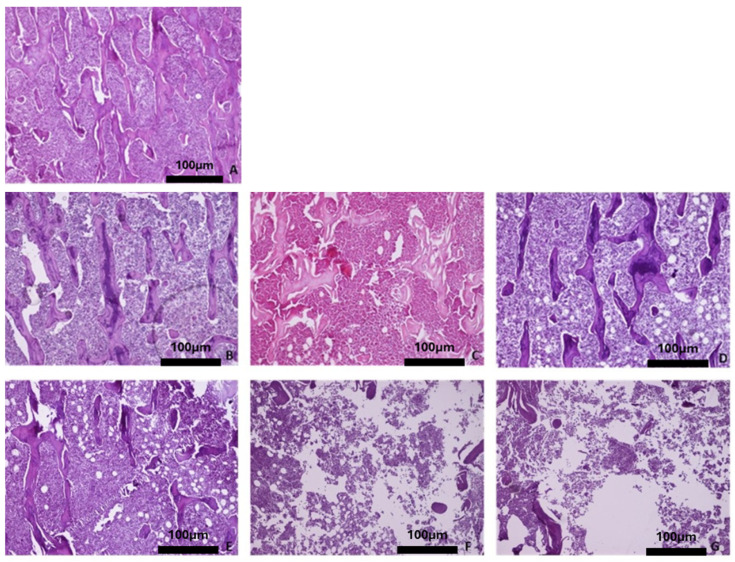
Representative histomicrographs of tibiae (hematoxylin–eosin staining; Obj ×10). Sections of the trabecular bone are depicted. In the OVX groups, the trabeculae are thinner, and the bone marrow cavities wider, compared to the control groups. (**A**) Baseline group, (**B**) control group 3 months, (**C**) control group 6 months, (**D**) control group 9 months, (**E**) OVX group 3 months, (**F**) OVX group 6 months, and (**G**) OVX group 9 months.

**Figure 6:**
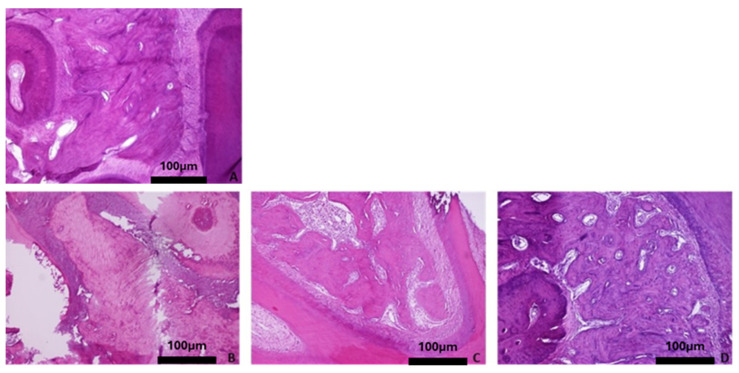

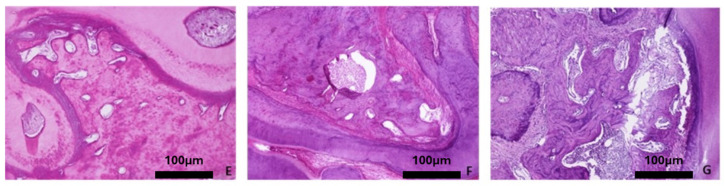
Representative histomicrographs of mandibles (hematoxylin–eosin staining; Obj ×10). No significant changes are depicted with aging or osteoporotic condition. (**A**) Baseline group, (**B**) control group 3 months, (**C**) control group 6 months, (**D**) control group 9 months, (**E**) OVX group 3 months, (**F**) OVX group 6 months, and (**G**) OVX group 9 months.

**Figure 7:**
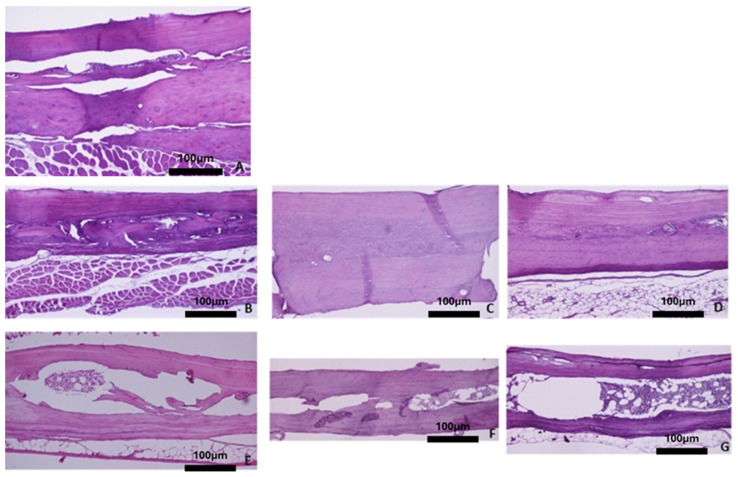
Representative histomicrographs of parietal bone (hematoxylin–eosin staining; Obj ×10). In the baseline group and the control groups, the cortical bone and part of the bone marrow are depicted. In the OVX groups, sections of the boundaries of the two cortical bones with the presence of hematopoietic marrow space in between are depicted. (**A**) Baseline group, (**B**) control group 3 months, (**C**) control group 6 months, (**D**) control group 9 months, (**E**) OVX group 3 months, (**F**) OVX group 6 months, and (**G**) OVX group 9 months.

Overall, the histological evaluation of the bones harvested from the OVX groups revealed trabecular bone reduction, while no significant differences in cortical bone between the OVX and Ctrl groups were observed. Regarding the baseline group, histological analysis of the femur showed dense trabecular bone with good coherence, while in the parietal bone the borders of the cortical bone were clear, and wide bone marrow cavities were observed. The characteristic alveolar bone structure was observed in the mandible and tibia (Figure 4A, Figure 5A, Figure 6A, and Figure 7A).

With advancing age, the four skeletal sites in the healthy rats did not show significant bone alterations with regards to qualitative bone characteristics (Figure 4B–D, Figure 5B–D, Figure 6B–D, and Figure 7B–D). Conversely, in the OVX3, OVX6, and OVX9 groups, the trabecular bone presented thinner, with widened bone marrow cavities (Figure 4E–G, Figure 5E–G, Figure 6E–G, and Figure 7E–G).

### 3.2. DXA Measurements

The DXA measurements performed for all examined anatomical sites are presented in Table 1.

#### 3.2.1. Mandible

Statistical analysis of the DXA measurements of the mandible revealed no significant differences between the OVX and Ctrl groups at 3, 6, and 9 months, although a statistically significant difference (*p* < 0.05) was observed between OVX9 and baseline. No statistically significant differences were observed at the different time points for the Ctrl groups, while the measurements in the OVX groups were decreased compared to baseline.

#### 3.2.2. Parietal Bone

Analysis of DXA measurements of the parietal bone indicated no statistically significant differences at the different time points for the Ctrl groups, while a significant decrease was observed in the OVX6 group compared to the baseline group (*p* < 0.05) and the OVX3 group (*p* < 0.05). Significant difference between the OVX and Ctrl groups was only found at 6 months (*p* = 0.012).

#### 3.2.3. Femur

A significant reduction in DXA measurements of the femoral diaphysis was observed in OVX rats compared to the baseline group at 3 (*p* < 0.05), 6 (*p* < 0.005), and 9 months (*p* < 0.005), while in the Ctrl groups a significant decrease was observed in comparison to the baseline group only at 3 (*p* < 0.005) and 6 months (*p* < 0.05). Moreover, DXA measurements of the femoral diaphysis showed significant reductions in the OVX groups compared to the Ctrl groups at 3 (*p* = 0.041), 6 (*p* < 0.001), and 9 months (*p* < 0.001).

The total femur DXA measurements presented statistically significant differences between the Ctrl and OVX groups at 6 (*p* = 0.003) and 9 months (*p* = 0.004), as well as between the OVX6 and OVX9 groups in comparison to the baseline group (*p* < 0.05).

#### 3.2.4. Tibia

DXA measurements of the proximal tibia revealed no statistically significant differences between the Ctrl groups (at 3, 6, and 9 months) and the baseline group. Significant reductions in the measurements were observed between the OVX 3 (*p* < 0.05), OVX6 (*p* < 0.05), and OVX9 groups (*p* < 0.005) and the baseline group. Additionally, statistically significant differences were found between the Ctrl and OVX groups at 3, 6, and 9 months (*p* < 0.001).

Regarding the DXA measurements of the total tibia, statistically significant differences were noted between the Ctrl and OVX groups at 6 (*p* = 0.037) and 9 months (*p* = 0.012), while the OVX9 group presented significantly reduced measurements compared to the baseline group (*p* < 0.005) and the OVX3 (*p* < 0.005) and OVX6 (*p* < 0.005) groups.

## 4. Discussion

The present pre-clinical study was designed in an attempt to elucidate the effects of experimental ovariectomy on different bones of the rat skeleton. We found that ovariectomy combined with a low-calcium diet in aged rats might lead to a reduction in the density of the parietal bone and mandible. The findings from this model can enhance the development of personalized biomimetic treatments that adapt to osteoporosis, optimizing therapeutic interventions based on the severity of bone loss at distinct time points.

As previously mentioned, a review of the current literature reveals heterogeneity with regards to the age of rats at ovariectomy. Rats are considered to be sexually mature at the age of approximately 2.5 months and reach skeletal maturity after the age of 10 months [22]. In particular, in order to fully investigate the rat as an experimental model of osteoporosis, 11-month-old rats were selected, their bone structure and bone density were assessed in four different regions (femur, tibia, parietal bone, and mandible), and a control group was included for better interpretation of the results. Since bone elongation occurs in the long bones of the rat skeleton throughout most of their lifespan, we included a baseline group to record the effects of growth on the skeleton and differentiate potential ovariectomy- and age-related bone changes. This baseline group represents day 0 of the study and is not incorporated in most of the available studies [51].

Furthermore, the outcomes were examined at three different time points: 3, 6, and 9 months after ovariectomy. It has been shown that changes in the trabecular bone in the mandible may be observed 3 months after ovariectomy, while the femoral neck, distal femur, and proximal tibia display significant bone loss within the first month after OVX [22,24,28]. The European guidelines for experimental testing of biomaterials require observation periods of at least 3 months (EN 30993-6) [58]. The evaluation of bone structure was performed with histological observation, and the measurement of bone density with DXA, which constitutes a reliable clinical method for investigating and classifying the severity of osteoporosis [59].

In the present study, ovariectomy was associated with a statistically significant increase in the rats’ body weight between the OVX and Ctrl groups at 3, 6, and 9 months. This finding is consistent with multiple studies [25,28,33,34,43,47,57,60,61,62,63]. It has been claimed that increased body weight may protect against osteoporosis, due to mechanical loading; meanwhile, a positive correlation may exist between body mass index and BMD in humans [64,65]. Moreover, reductions in estrogen levels (OVX groups) resulted in decreases in bone density, which varied among the different skeletal sites. Regarding the bone density measurements in the Ctrl groups, our results indicate that the parietal bone, mandible, total tibia, proximal tibia, and total femur do not present significant age-related changes in bone density. These observations are in agreement with previous publications that found no significant changes in bone density associated with aging at the aforementioned skeletal sites [24,28,34,35].

The OVX rat model has been established for the evaluation of changes in the alveolar bone [25,66]. The mandible seems to be less sensitive to ovariectomy due to its morphological and embryological characteristics, and possibly because of the effect of masticatory forces [41,47,60]. It has been claimed that although the muscular forces applied during mastication prevent bone loss in OVX rats, they may not activate bone remodeling and mineralization, because of estrogen deficiency [41,45,46]. Multiple studies have shown statistically insignificant changes in the bone density of the mandible between OVX and control rats aged from 4 to 28 weeks [37,41,42,67]. In our study, the rats were older (11 months old at ovariectomy), and significant differences were observed in the DXA measurements of the mandible between the baseline and OVX groups at 9 months. This finding may indicate that advanced rat age, in combination with OVX, may induce bone changes similar to osteoporosis in the mandible.

According to our results, the DXA measurements of the parietal bone showed a significant reduction in bone density only at 6 months after ovariectomy compared to the control group at 6 months and the baseline group. This result may indicate that a longer time period after ovariectomy is required in order for the parietal bone in the OVX groups to present significantly reduced bone density compared to the control and baseline groups. Interestingly, no significant differences in DXA measurements were identified between the OVX and control groups at 9 months, which may be explained by the age-related bone loss in the control group Moreover, in the late stages of estrogen deficiency, hypermineralization of the existing bone occurs, thus leading to increased mean mineral density [68]. Limited studies reporting on the effects of ovariectomy on the parietal bone are available in the literature. It has been found that trabecular bone responds more rapidly to ovariectomy than cortical bone, which occupies the largest part of the ROI in the parietal bone [24,28]. This may explain why DXA may not recognize changes in the trabecular bone of the parietal bone 3 months after ovariectomy. Liu et al. examined the effects of ovariectomy on the structure and density of the parietal bone in 6-month-old rats, and they did not observe significant changes between the test and control groups up to 36 months after ovariectomy [24]. Although further studies are needed, it could be claimed that ovariectomy at a more advanced age leads to osteoporotic characteristics in the parietal bone.

The DXA measurements of the total femur and total tibia revealed significant differences between the OVX and control groups at 6 and 9 months. Existing studies that examined rats undergoing ovariectomy at the ages of 3, 5, and 6 months revealed a significant decrease in total femur BMD in the OVX group compared to the control group 2 to 5 months after ovariectomy [37,69,70]. DXA measurements in the total tibia of the OVX group at 9 months were also significantly reduced compared to the baseline and OVX groups at 3 and 6 months. This finding indicates that 9 months after ovariectomy, an osteoporotic condition is clearly induced in the total tibia, which may be used in studies examining the effects of various treatments on bone density.

In the femoral diaphysis and proximal tibia, statistically significant changes in bone architecture and bone density were observed in the OVX groups at 3, 6, and 9 months in comparison to the baseline and control groups. These findings are in accordance with previous studies suggesting that OVX leads to an early decrease in bone density and alteration of the bone micro-architecture at these skeletal sites [24,30,35,48,60,61,71,72,73]. The DXA measurements of the femoral diaphysis were significantly higher in the baseline group compared to the control groups at 3 and 6 months after ovariectomy, which may indicate bone loss due to aging. As previously mentioned, no age-related bone loss was observed in the proximal tibia; an osteoporotic-like condition was induced soon after ovariectomy (at 3 months) and persisted for at least 9 months. Hence, within the limitations of this study, it may be suggested that the proximal tibia in rats undergoing OVX at 11 months would be the most appropriate skeletal site for use in studies exploring the outcomes of dental implantation and bone regeneration procedures in osteoporotic conditions.

The rat model is commonly used in experimental studies evaluating osseointegration and bone regeneration. Nonetheless, the ideal experimental model has not yet been found, possibly because bone changes induced by osteoporosis arise naturally only in humans and may be experimentally induced in animals [3]. Furthermore, an increase in body weight after OVX results in an increase in the mechanical load, which could protect OVX rats against bone loss; therefore, histologically and radiographically, the bone changes present more similar to those of osteopenia than of osteoporosis [16].

One limitation of this study is the absence of a sham-operated control group. Healthy, non-operated controls were included to represent normal bone architecture under standard dietary conditions. The inclusion of a sham group would have allowed for a more precise distinction between the effects of ovariectomy and those associated with surgical stress or anesthesia. Moreover, due to ethical considerations, we did not include additional animals, which would have accounted for potential losses. Two rats died in the present study: one from the control group and one from the OVX group.

The bone changes observed in the OVX rat model reflect the clinical presentation of postmenopausal osteoporosis and offer an ideal setting for testing bioactive scaffolds—for instance, for hard tissue regeneration. The ovariectomized rat model presented in this study may serve as a valuable tool for the pre-clinical evaluation of scaffold materials with osteoinductive and osteoconductive properties.

In conclusion, the findings of the present study might imply that ovariectomy combined with a low-calcium diet in older rats (11 months old), rather than those used in most of the published studies (3 months old), could lead to bone density reductions in the mandible, parietal bone, femoral diaphysis, proximal tibia, total femur, and tibia. Among the aforementioned skeletal sites, the proximal tibia of rats undergoing OVX at 11 months seems to present the most appropriate osteoporotic response for use in pre-clinical studies. Overall, the results emphasize the importance of site-specific biomimetic approaches in bone tissue engineering. Future biomaterials should be tailored not only to match the structural composition of the bone but also to adapt to site-specific mechanical and biochemical cues, ensuring enhanced integration and longevity in clinical applications such as orthopedic and maxillofacial reconstructions. Although the rat model is commonly used in the research of bone regeneration and osseointegration, data from animal models should be cautiously processed, because their response to estrogen deficiency is not identical to the human one.

## Figures and Tables

**Table 1:** Analysis of DXA measurements.

Site	Baseline	Groups	3 Months	6 Months	9 Months	*p*-Value _over time_	*p*-Value _interaction_
Mandible	0.297 ± 0.025	Ctrl	0.253 ± 0.044	0.274 ± 0.023	0.270 ± 0.035	0.174	0.598
	0.297 ± 0.025	OVX	0.265 ± 0.019	0.262 ± 0.011	0.256 ± 0.006 ^a^	0.028	
	1.000	P-	0.584	0.224	0.430		
Parietal bone	0.082 ± 0.044	Ctrl	0.128 ± 0.072	0.085 ± 0.059	0.078 ± 0.012	0.507	0.199
	0.082 ± 0.044 ^b^	OVX	0.087 ± 0.034 ^b^	0.017 ± 0.011	0.068 ± 0.01 ^c^	<0.001	
	1.000	P-	0.280	0.012	0.206		
Femoral diaphysis	0.300 ± 0.038	Ctrl	0.245 ± 0.017 ^d^	0.255 ± 0.015 ^a^	0.261 ± 0.026	0.009	0.01
	0.300 ± 0.038	OVX	0.207 ± 0.035 ^a^	0.191 ± 0.009 ^d^	0.162 ± 0.025 ^d^	0.001	
	1.000	P-	0.041	<0.001	<0.001		
Total femur	0.242 ± 0.018	Ctrl	0.234 ± 0.047	0.246 ± 0.021	0.252 ± 0.020	0.767	0.021
	0.242 ± 0.018	OVX	0.200 ± 0.029	0.204 ± 0.006 ^a^	0.177 ± 0.039 ^a^	0.002	
	1.000	P-	0.186	0.003	0.004		
Proximal tibia	0.328 ± 0.062	Ctrl	0.286 ± 0.023	0.278 ± 0.049	0.252 ± 0.020	0.132	0.029
	0.328 ± 0.062	OVX	0.215 ± 0.014 ^a,e^	0.222 ± 0.016 ^a,e^	0.165 ± 0.01 ^d^	<0.001	
	1.000	P-	<0.001	<0.001	<0.001		
Total tibia	0.209 ± 0.014	Ctrl	0.191 ± 0.022	0.202 ± 0.010	0.207 ± 0.023	0.316	0.007
	0.209 ± 0.014 ^e^	OVX	0.195 ± 0.029 ^e^	0.190 ± 0.007 ^e^	0.163 ± 0.005	<0.001	
	1.000	P-	0.776	0.037	0.012		

Data are presented as the mean ± standard deviation: ^a.^ vs. baseline *p* < 0.05; ^b.^ vs. 6 months *p* < 0.05; ^c.^ vs. 6 months *p* < 0.005; ^d.^ vs. baseline *p* < 0.005; ^e.^ vs. OVX9 *p* < 0.005.

## Data Availability

The data presented in this study are available on request from the corresponding author.

## Abbreviations

The following abbreviations are used in this manuscript:

OVX	Ovariectomized
Ctrl	Control
FDA	Food and Drug Administration
DXA	Dual-energy X-ray absorptiometry
BMD	Bone mineral density
ROI	Region of interest
CI	Confidence interval
SD	Standard deviation
